# Characterization of pharmaceutical medication without a medical prescription in children before hospitalization in a resource-limited setting, Cameroon

**DOI:** 10.11604/pamj.2018.30.302.16321

**Published:** 2018-08-31

**Authors:** Calixte Ida Penda, Else Carole Eboumbou Moukoko, Julien Franck Ngomba Youmba, Emmanuel Mpondo Mpondo

**Affiliations:** 1 Department of Clinical Sciences, Faculty of Medicine and Pharmaceutical Sciences, University of Douala, Douala, Cameroon; 2 Department of Pediatrics, Laquintinie Hospital of Douala, Cameroon; 3 Department of Biological Sciences, Faculty of Medicine and Pharmaceutical Sciences, University of Douala, Cameroon; 4 Malaria Research Service, Centre Pasteur Cameroon, Yaoundé, Cameroon; 5 Department of Pharmaceutical Sciences, Faculty of Medicine and Pharmaceutical Sciences, University of Douala, Cameroon

**Keywords:** Therapeutic itineraries, children, self-medication, hospitalization, Cameroon

## Abstract

**Introduction:**

The use of different therapeutic approaches is common among sick children in Cameroon. The main objective of this study was to characterize the use of non-prescription drugs and describe the therapeutic itineraries of sick children before admission to the hospital.

**Methods:**

A cross-sectional and prospective study was conducted from January to May 2017. A closed-ended questionnaire (CEQ) consisting of one or several response options was administered to the parents/guardians of the children on admission to the hospital in the pediatric ward of the Laquintinie Hospital in Douala (LHD) and the Cité des Palmiers District Hospital (CPDH) of the city of Douala. Inclusion of participants was made consecutively for adolescents who gave their consent and parents or guardians who signed the informed consent for all children. The confidentiality of the data was ensured by the replacement of the names by codes.

**Results:**

Overall, 295 hospitalized children were included with an average age of 3.1 (SD: 3.3) years in the study. More than half of these children (58.6%) came from LHD. More than 90% of parents had at least one therapeutic recourse (TR). The ratio of boys to girls 3/1. Self-medication (74.1%) and medical consultation (16.9%) were the main therapeutic paths in 1^st^ recourse. The medical consultation (80.2%) and the pharmaceutical advice (16.9%) were used frequently in 2^nd^ recourse. The mean lapse time to see a medical professional was 2.7 days (min-max: 0-14 days). The main symptoms associated with TR were fever (76.6%), vomiting (24.7%) and diarrhea (22.7%). The most frequently used drugs were Analgesics/antipyretics (47.6%), antimalarials (15.0%) and antibiotics (10.2%) and the family medicine box was the highest source of drugs.

**Conclusion:**

Self medication remains the first therapeutic path, followed by medical consultation as second therapeutic path taken when the disease is perceived as serious.

## Introduction

Therapeutic itineraries (TI) have become considerably diversified with the transition from traditional medicine to modern medicine under the influence of various religions in Africa. The supply of healthcare systems has expanded but with limited and unequal access to care [1-3]. TIs refer to the pathways that patients and their families follow and the therapeutic options or remedies they choose depending on the context in which they evolve. TI can be in a public or private health facility, formal or non-formal, modern or traditional medicine, and the use of healthcare services as first or second level according to the health pyramid [4-9]. The combined use of modern medicine and alternative medicines are frequent. Previous studies conducted on the nature and determinants of these treatment options have identified several factors (environmental, socio-cultural and economic, demographic issues, diversity of supply and accessibility to different health centers) influencing the use of services [10-18]. In Africa, self-medication (SM) is the first therapeutic remedy. It is defined as the use of drugs to treat a pathological situation, real or imagined without prior medical consultation on the indication, dosage and duration of treatment [19]. SM involves the use of medication by the consumer to treat self-recognized disorders or symptoms, or the intermittent or continuous use of a medication prescribed by a physician for chronic or recurring illnesses or symptoms by family members, especially in children or the elderly [20]. Barbieri reported that “behavioral codes for a disease would be directly determined by the recognition and classification of symptoms” [21].

On the contrary, the use of healthcare services is seen as a constraint imposed by the severity of the disease in children [22], moreover the perception of the severity of the disease contributed in 71% of the cases in the use of the self-medication [10]. The interest of consulting a doctor is perceived as essential by the patient or their guardian only in case of presumed gravity of the disease, or after a failure of a drug (pharmacy, road side or even traditional medication) often badly used. In the case of children, TI and the risks related to pediatric self-medication have been the subject of few studies [23-26]. Several studies in Europe, India and Africa on self-medication have identified the transient nature of the disease [10,27,28], limited financial resources [22] and high cost of care, as being the main reasons for justifying SM [29]. Other factors, such as education, sex, socio-economic status, availability of drugs and socio-cultural perception of the disease, also influenced the population's response to the disease [10,19]. Only two studies of care seeking behavior and household medications, conducted in 2006 and 2011 based on data from the 2011 DHS-MICS [30], indicated that self-medication appears as a first-line therapeutic itinerary. In first intention, 60% of children aged 5 to 14 and 54% of children under 5 had self-medication in Cameroon [10,31]. The continuous diversity of care supply and ongoing epidemiological transition in Cameroon are calling for more recent data. The aim of the study was to characterize the use of non-prescription drugs through the analysis of therapeutic itineraries taken by sick children before admission to a hospital.

## Methods

**Study setting:** The study was conducted in Douala, a town located in the Littoral region of Cameroon. Two health facilities were used. The pediatric emergency unit in Laquintinie Hospital Douala (PEU/LHD) which is a reference hospital (higher level of the health pyramid) located in the Deido Health district of Douala I and the pediatric ward in « Cité des Palmiers » District Hospital (CPDH) which is a hospital (lower level) located in the health district of Douala V.

**Study population, overall study design:** A cross-sectional and prospective study was conducted among children and adolescents aged 0 to 18 years before admission to 2 health facilities in the city of Douala selected by convenience. Inclusion of participants was made consecutively for adolescents who gave their consent and parents or guardians who signed the informed consent for all children. Children/ adolescents with incomplete records and those hospitalized at birth were excluded from the study. The confidentiality of the data was ensured by the replacement of the names by codes. After a one-week pre-test on 30 parents/guardians, a questionnaire was administered to parents to assess: i) understanding and acceptability of participants and ii) standardized and harmonized data collection at both sites. Interview questions were formulated to avoid influencing participants in their responses. These were open-ended (OEQ) and closed-ended (CEQ) questions with one or more response choices. The pre-test helped to better organize the questionnaire We used data sources from EDS -IV-MICS, on the proportion of children aged 0 to 14 years hospitalized in Cameroon who were sick/injured in 2011. About 170 children and adolescents were needed according to the prevalence of 12.7% (Accuracy 10%, alpha 5%, power 80%) [30,32]. A total of 314 children and adolescents were selected.

**Data collection and study variables:** A semi-structured interview guide was developed to list all therapeutic options (TOs), patient itinerary and reasons for choosing it. To define patients' TO, we used four key variables as follows: “Recourse to self-medication” based on modern or traditional medicines available at home, bought in pharmacies or on the street or provided by a traditional healer. “Medical recourse” as any use of a health service (public or private such as a hospital, health center, and clinic). “Pharmaceutical advice” as advice given by a health professional (pharmacist or clerk) on a health problem to a patient before the purchase or not of a drug with or without a doctor's prescription “Traditional medicine as any medication based on traditional treatments or the use of a traditional healer (recognized person using knowledge, skills and practices based on theories, beliefs and empirical experiences to keep humans healthy).

**Definition of operational terms:** Therapeutic recourse was defined as the various requests for care which a person can make to a specific group of people or an institution during a morbid condition. These recourses include self-medication, traditional medicine and modern medicine. This same definition was proposed in 1978 by Kleiman in the classification of health care systems and was updated by Akoto et *al* [33,34]. Medical prescription is an act of prescribing treatment on a prescription, after having made a diagnosis. The prescription may concern drugs, medical devices, biological or radiological examinations. The right prescription recommended in Cameroon is done by doctors, midwives, dentists and nurses as part of the delegation of tasks. We administered a questionnaire to collect information on socio-demographic characteristics of children/adolescents (age, sex, place and year of birth) and of parents/guardians (age, sex, level of education, occupation and family economy context, perception of the disease and place of residence to identify accessibility to a healthcare facility, the health related cost and the reasons for the choice of TO and TI). We also collected information that could justify the choice of TO and TI to identify accessibility to a healthcare facility and health related cost. Starting from simple cross tabulation of the generated variables, we analyzed the TI of sick children/adolescents (self-medication, formal care, traditional medicine) during a given episode of illness before ad-mission to pediatric ward. From simple tabulation and cross-matching of variables, we were able to picture the TI of sick children/adolescents as the sequence (traditional medicine, self-medication, formal care) within a given disease episode before admission to pediatric ward. The interviewer also collected information on drugs consumed, the time-to-medical intervention. Other variables were accessed from the clinical records, such as child's initial disorders and symptoms, nature of the disease (acute or chronic), the length of hospital stay and the clinical outcome of the patient. Information about drugs consumed was classified as follows: 1) Drugs from the pharmacy: modern medicines available at home or bought at the pharmacy; 2) Drugs in the street: modern medicines bought at the market/street; 3) Traditional drugs: traditional remedies. The time-to-medical intervention was defined as the median length of homestay (defined as the number of days between the onset of clinical symptoms at home and admission to one health facility).

**Data analysis:** Categorical variables were expressed in terms of frequency and numerical variables were pre-sented as averages +/- SD or 95% CI (95% confidence interval) if they were normally distribut-ed. To compare the proportions, we used Fisher's Exact test. Numeric values were compared using the Wilcoxon U test. All statistical analyzes were done using the Stata software (version 11 SE). Only p-values <0.05 were considered significant in all analyzes.

**Ethical aspects:** The Institutional Review Board of the University of Douala (IRB/UD) approved the study N°CEI/UDo/961/16/2017/T and administrative authorization for research from each health facilities (N°0985/AR/MINSANTE/DHL/CM from the LHD and N°0055/AR/MINSANTE/HDCP) was obtained.

## Results

**Figure 1:**
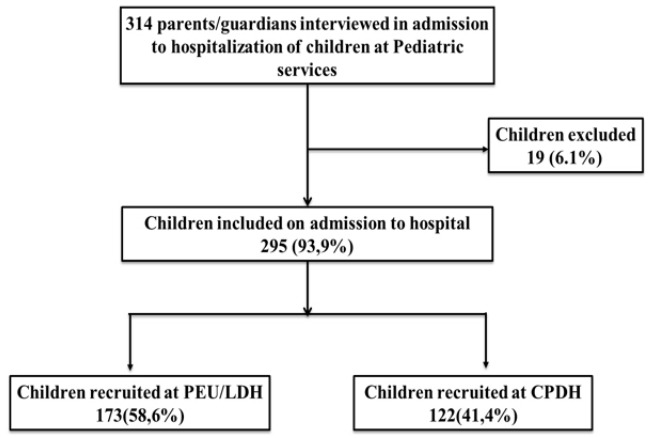
Flow diagram of inclusion and exclusion process of selected children in the study

**Figure 2:**
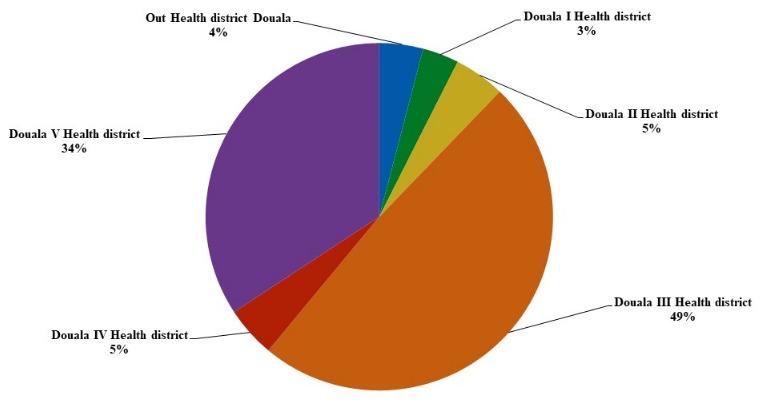
Places of residence of patients

**Characteristics of study population:** In total, 501 children/adolescents were admitted to health facilities during the survey, 314 of the 400 children/adolescents who met the inclusion criteria, agreed to participate in the study, of which 19 had incomplete clinical record. The analysis involved a total sample of 295 children/adolescents (58.88%) prior to their admission at the pediatric units (Figure 1). The main characteristics of children/adolescents and their parents/guardians are presented in Table 1. The mean age of children/adolescents was 3.1 years (min: 1 month- max: 15 years) and was 2.5 times higher in children from CPDH than in children from PEU/LHD. Boys accounted for more than half of all children and no significant differences were observed between girls and boys and between study locations for the other variables studied. The children were accompanied by parents who were mostly Christian women with no professional activity. Most parents/guardians of children admitted to LHD had no professional activity, while most parents of children included in CPDH had formal employment (p = 0.024). The distribution of children/adolescents according to the parents/guardians' residence, are shown in Figure 2. Almost all patients (96%) came from the 6 health Districts of the Douala city, and particularly in the 3^th^ and 5^th^ district which represented 83% of residences and only 3% of the children came from the 1^st^ health district of Douala where the reference hos-pital is located. According to health expenses, less than 9% (26/295) of patients had health insurance from their parents/guardians. Most of the patients (14/26, 53.8%) with health insurance came from CPDH.

**Table 1:** General socio-demographic characteristics of children and their parents/guardians

	Girls	Boys	Total
N (%)	130 (44.1)	165 (55.9)	295 (100)
**Mean age (+/-SD), years**	3.2 (3.5)	2.9 (3.2)	3.1 (3.3)
≤ 4 years	88 (67.7)	128 (77.6)	216 (73.2)
≥ 5 years	42 (32.3)	37 (22.4)	79 (26.8)
**Sex of parent/guardian**			
Female	123(94.6)	155 (93.9)	278 (94.2)
Male	7(5.4)	10 (6.1)	17(5.8)
**Religion of parent/guardian**			
Christian	125 (96.1)	161 (97.6)	286 (96.9)
Muslim	5 (3.9)	4 (2.4)	9 (3.1)
**Education Level of parent/guardian**			
None	4 (3.1)	4 (2.4)	8 (2.7)
Primary	14 (10.7)	19 (11.5)	33 (11.2)
Secondary	84 (64.7)	107 (64.9)	191 (64.8)
University	28 (21.5)	34 (20.7)	62 (21.0)
Unknown	0 (0.0)	1 (0.3)	1 (0.3)
**Occupation of parent/guardian**			
Student	11 (8.4)	10 (6.1)	21 (7.1)
Unemployed	79 (60.8)	82 (49.8)	161 (54.6)
Public sector	5 (3.5)	11 (6.7)	16 (5.4)
Private sector	10 (7.7)	20 (12.1)	30 (10.2)
Informal sector	25 (19.2)	42 (25.4)	67 (22.7)

### Therapeutic itinerary of hospitalized patients

*Time-to-medical intervention*: the time to see a health professional was 2.7 days (inter-quartile range ((IQR) =1-3) and was lower in girls (2.5 with min-max: 0-14 days) compared to boys (2.9 days with min-max: 0-20) but this difference was not statistically significant (p=0.577). According to age groups and health facility, no statistically significant difference was found.

**Figure 3:**
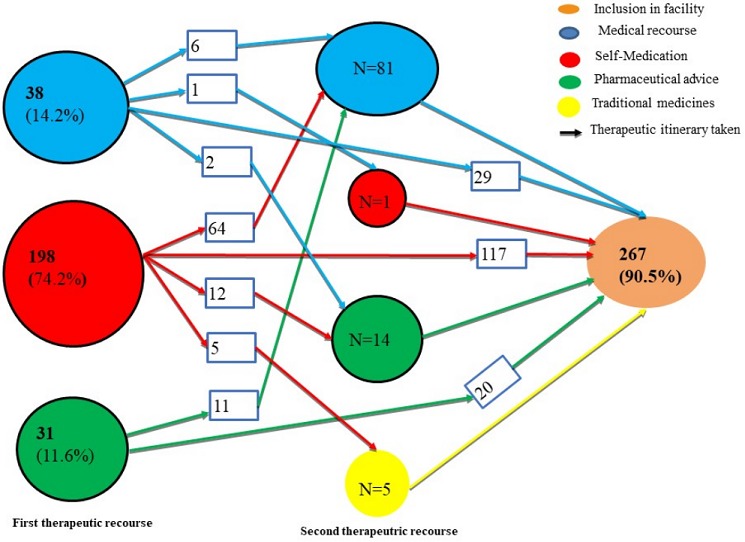
Therapeutic itineraries followed by parents/guardians of children before their admission in hospitalization at health facilities

*Therapeutic options and therapeutic itineraries of patients*: Table 2 describes the patients' therapeutic itineraries prior to admission to pediatric ward according to gender and health facility. Overall, TR were more frequent among the boys (93.3% vs 86.9% for girls, p=0.048). The difference remained significant when we compared the number of patients with one or two TR according to gender (OR=1.78, CI95%=1.07-2.99, p=0.026). We found also that the TOs varied according to gender. Self-medication was the initial most widely used option (74.2%), followed by medical recourse for girls (19.5%) and a hospital for boys (14.9%). Of the 69 patients surveyed using formal health care service, medical recourse represented 55% of TOs, more among girls (73.3%) than boys (41%) compared to pharmaceutical recourses. This difference was statistically significant (OR=3.95, 95%CI: 1.41-11.07, p = 0.009). The frequencies between four groups of TO reported by parents/guardians are shown in Table 3. Overall, the TOs were significantly varied in search of care. The various TOs were used either exclusively or in combination (i.e. sequentially) by the different health facilities and parents/guardians. Of the 267 patients who used a TR before admission, 166 (62.6%) had just one TI and 101 (37.8%) had two TIs. Overall 74.2% of patients had self-medication as first TR and 14.2% undertook medical recourse. We also reported that 101 of the 267 patients (37.8%) who used TR shifted from first to second TR. Most of the parents/guardians who attended the CPDH used one TI compared to those who attended the PEU/LHD, who had used two TI before admission of children/adolescents to health facilities (p=0.0001). For up to 80% of these 101 patients, self-medication care was the initial TO. The shift from self-medication as 1^st^ TO to medical recourse as second option was 79.0% (64/81), compared to 11.1% (1/9) in the opposite direction; this difference was not statistically significant (p =0.099). Similar to second TOs, medical recourse was the most widely used (80.2%), followed using a Pharmaceutical recourse. Parents/guardians who admitted using traditional medicine accounted for less than 5% of patients' itineraries in the 2^nd^ TO. Traditional medicine was not used as an initial TO Figure 3 represents the 10 different therapeutic itineraries described by parents/guardians before their child's admission. Of the 295 patients included, the majority (267/295, 90.5%) of patients had at least one therapeutic recourse (TR) prior to the admission to health facilities for their children. After being in the first health facility, 29 out of the 38 children went directly to one of the inclusion sites while 6 out of them went to a second health facility in second intention before admission in the inclusion sites. Out of the 198(74.2%) children who had self-medication, the majority of them (117) went directly to the sites of inclusion for admission, 64 of them were consulted in another health facility while only 5 were consulted by a traditional healer before admission. Among the 31 children who received pharmaceutical advice as first TO, 11 children went to another health facility before their admission and 20 children went directly to the sites of inclusion as second intention.

**Table 2:** Patient's therapeutic recourses according to gender and health facilities

Number of therapeutic recourses	Gender				Health facilities			
	Girls	Boys	Total	p	PEU/LHD	CPDH	Total	p
	n=130	n=165	n=295		n=173	n=122	n=295	
No recourse	17 (13.1)	11 (6.7)	28 (9.5)	1	16 (9.2)	12 (9.8)	28 (9.5)	1
First recourse	79 (60.8)	87 (52.7)	166 (56.3)	0.140	69 (39.9)	97 (79.5)	166 (56.3)	0.086
Second recourse	34 (25.1)	67 (40.6)	101 (34.2)	0.009	88 (50.9)	13 (10.7)	101 (34.2)	0.001

PEU: pediatric emergency unit, LHD: Laquintinie Hospital of Douala, CPDH: Cite Palmier District Hospital. Data are number and/or proportion (%) of patients’ patterns according to gender and health facilities. Unless otherwise indicated

**Table 3:** Patients' therapeutic itineraries according to therapeutic recourse and health facilities

Therapeutic recourses								
Therapeutic itineraries	1^st^ recourse				2^nd^ recourse			
	PEU/LHD n=157	CPDH n=110	Total n= 267	*p*	PEU/LHD n=88	CPDH n=13	Total n=101	*p*
Medical recourse	35 (22.3)	3 (2.7)	38 (14.2)	1	72 (81.8)	9 (69.2)	81 (80.2)	1
Self-medication	104 (66.2)	94 (85.5)	198 (74.1)	10^-4^	1 (1.1)	0 (0.0)	1 (1.0)	/
Pharmaceutical Advice	18 (11.5)	13 (11.8)	31 (11.7)	10^-3^	10 (11.4)	4 (30.8)	14 (13.9)	0.431
Traditional medicine	0 (0.0)	0 (0.0)	0 (0.0)	/	5 (5.7)	0 (0.0)	5 (4.9)	/

PEU: pediatric emergency unit, LHD: Laquintinie Hospital of Douala, CPDH: Cite Palmier District Hospital. Data are number and/or proportion (%) of the patients according to each of the variables indicated

**Choice of the therapeutic option:** The reasons given for the choice of the option and the therapeutic recourse are shown in Table 4. The perception of mild illness and treatment knowledge reported by parents/guardians were sig-nificantly associated with the choice of non-formal health care in the 1^st^ TR (p=0.0001). Mild illness was predominant (55.7%) in the 1^st^ TR while persistence (52.2%) and severity (26.5%) of the disease were the main reasons why patients/guardians had used the medical staff of the health facilities, more represented in the 2^nd^ TR (81/101, 80.2%). Other reasons reported for use 2^nd^ TR were the recurrence of symptoms despite the treatment taken during the first therapeutic recourse.

**Table 4:** Factors affecting therapeutic recourses

Therapeutic recourses									
1^st^ recourse					2^nd^ recourse				
Factors	Self-Medication	Pharmaceutical Advice	Medical recourse	Total	Self-medication	Pharmaceutical advice	Medical recourse	Traditional healer	Total
Mild illness	156	26	0	182 (55.7)	1	6	0	0	7 (6.2)
Insufficient funds	17	3	0	20 (6.1)	0	0	0	0	0
Lack of time	3	2	0	5 (1.5)	0	0	0	0	0
Knowledge ontreatment	29	0	0	29 (8.9)	0	0	0	1	1 (0.9)
Advice from a friend	9	0	1	10 (3.1)	0	0	0	3	3 (2.7)
Disease requiringmedical consultation	0	3	22	25 (7.6)	0	1	10	0	11 (9.7)
Disease severity	0	0	0	0	0	3	27	0	30 (26.5)
Persistent disease	0	0	0	0	0	5	54	0	59 (52.2)
Others	32	2	22	56 (17.1)	0	0	0	2	2 (1.8)

**Manifestations, diagnosis and clinical evolution:** Clinical manifestations reported by responders for 1^st^ TR are represented in **Table 5**. Fever was the predominant symptom at the origin of the use of self-medication (82.8%), followed by vomiting (19.7%) and diarrhea (19.7%), cough (12, 6%), rhinorrhea (9.1%) and headache (9.1%) respectively. The prevalence of patients reported vomiting differed significantly according to the choice of formal or informal recourse (p=0.010). The mean time-to-hospitalization was statistically significantly higher for patients with 2 TR (6.75 days, IQR: 4-8) compared to patients with 1 TR (4.5 days, IQR: 3-5) (p=0.0001). Globally, 93.2% of patients were cured after their hospitalization. The respective cure rates of patients who used a single therapeutic recourse were 95.7% (112/117) for self-medication, 95.0% (19/20) for medical consultation and 75.8% (22/29) for hospital referral after hospitalization. Many patients who used two TRs were declared cured, except for a patient referred to another health facility and discharged against medical advice.

**Table 5:** Common signs and symptoms and therapeutic options of study population

	Informal medicine	Formal medicine		Total
Signs and Symptoms	Self-medication N=198	Pharmaceutical sdvice N=31	Medical recourse N=38	267
Fever	164 (82.8)	21 (67.7)	28 (73.7)	213 (79.8)
Asthenia	14 (7.1)	1 (3.2)	2 (5.3)	17 (6.4)
Fainting	0 (0.0)	0 (0.0)	1 (2.6)	1 (0.4)
Loss of appetite	12 (6.1)	3 (9.7)	3 (7.9)	18 (6.7)
Weight loss	0 (0.0)	1 (3.2)	1 (2.6)	2 (0.7)
Convulsion	2 (1.0)	0 (0.0)	3 (7.9)	5 (1.9)
Headaches	18 (9.1)	3 (9.7)	0 (0.0)	21 (7.9)
Cough	25 (12.6)	5 (16.1)	6 (15.8)	36 (13.5)
Dyspnea	3 (1.5)	0 (0.0)	2 (5.3)	5 (1.9)
Rhinorrhea	18 (9.1)	5 (16.1)	4 (10.5)	27 (10.1)
Diarrhea	39 (19.7)	8 (25.8)	15 (39.5)	62 (23.2)
Vomiting	39 (19.7)	11 (35.5)	13 (34.2)	63 (23.6)
Abdominal pain	22 (11.1)	3 (9.7)	1 (2.6)	26 (9.7)
Others*	23 (11.6)	9 (29.0)	4 (10.5)	36 (13.5)

Data are number and/or proportion (%) of patients’ patterns according to therapeutic option. Others

* : includes nausea, conjunctivitis, rash, wounds, joint pain, mumps, dizziness, bleeding, constipation, pallor, inflammation.

### Administered drugs

*Origin, classification and reading of drug information leaflets*: the Table 6 describes the treatment history of children prior to admission at the pediatric units. The mean number of drug units taken per child was 1.7 (1-6 units). Medications consumed without medical prescription were 75.26% under self-medication and 24.74% under pharmaceutical advice. The main origin of the medicines consumed as self-medication before admission to the hospital came from the family pharmacy box (64%), followed respectively by drugs bought in pharmacies (22%) and those purchased in the street (14%). Unlisted medications consumed as self-medication accounted for 2/3 of medicines administered without medical advice against 1/4 for those on list 1 and 9% on list 2. In addition, 80.7% (188) of the drugs on the list 1 were used as self-medication and the rest under pharmaceutical advice. The high prevalence of medications taken as self-medication was also observed in list 1 (68.3%) and list 2 (62.8%). The rate of reading of drug information leaflets was higher when the parent/guardian had received pharmaceutical advice (74.5% vs 65.4% for self-medication).

**Table 6:** The use of multi-drug therapy in the study population

Drugs	Frequency	ID*	DI^$^	CI^£^
	N=380 (%)	N=112 (%)	N=15 (%)	N=5 (%)
**Origin of drug**				
Family pharmacy box	182 (47.89)	68 (60.71)	13 (86.67)	3 (60.00)
Pharmacy	157 (41.32)	19 (16.97)	2 (13.33)	2 (40.00)
Drugs from the street	41 (10.79)	25 (22.32)	0 (0.00)	0 (0.00)
**Type of therapeutic recourse**				
Self-Medication	286 (75.26)	104 (92.86)	15 (100.00)	4 (80.00)
Pharmaceutical advice	94 (24.74)	8 (7.14)	0 (0.00)	1 (20.00)
**Classification according to list**				
List 1	104 (27.37)	41 (36.61)	0 (0.00)	0 (0.00)
List 2	43 (11.32)	11 (9.82)	1 (6.67)	2 (40.00)
Unknown List	233 (61.31)	60 (35.57)	14 (93.33)	3 (60.00)
**Therapeutic Classes**				
NSAI^§^	3 (3.80)	2 (1.80)	1 (6.70)	2 (40.00)
SAI^&^	2 (0.50)	0 (0.00)	0 (0.00)	0 (0.00)
Antianemic	9 (2.40)	0 (0.00)	0 (0.00)	0 (0.00)
Antiasthenic	7 (1.80)	0 (0.00)	0 (0.00)	0 (0.00)
Antibiotic	39 (10.30)	18 (16.10)	0 (0.00)	0 (0.00)
Antidiarrheal	8 (2.10)	1 (0.90)	0 (0.00)	0 (0.00)
Antiemetic	14 (3.70)	7 (6.30)	0 (0.00)	0 (0.00)
Antifungal	10 (2.60)	3 (2.70)	0 (0.00)	0 (0.00)
Antihelminthic	21 (5.50)	2 (1.80)	0 (0.00)	0 (0.00)
Anti-malarial	57 (15.00)	20 (17.90)	0 (0.0)	0 (0.0)
Analgesic/Antipyretic	181 (47.60)	49 (43.70)	8 (53.30)	0 (0.00)
Antitussif	8 (2.10)	3 (2.70)	0 (0.00)	3 (60.00)
Antirhinorrhea	17 (4.50)	6 (5.30)	6 (40.00)	0 (0.00)
Others	4 (1.10)	1 (0.90)	0 (0.0)	0 (0.00)

Data are number and/or proportion (%) of indicated variable.

ID^*^: Incorrect dosage.

I^$^: Drug interaction.

CI^£^: Contraindication.

NSAI^§^: Non-steroidal anti-inflammatory.

SAI^&^: steroidal anti-inflammatory.

*Therapeutic class, drug interaction and misuse of medications consumed*: of the 380 drug units, 132 (34.74%) were misused (**Table 6**). Antibiotics, analgesics and anti-malarials were the therapeutic classes where most of the misuse was found. Of the 132 misused drugs, dosage errors were observed in 84.8% of cases, 11.3% for drug interactions and 3.8% for contraindications. Drug interactions have been found in paracetamol based combination therapies and contraindications were most common in younger children. The misused drugs were mainly used in self-medication (116/124, 93.55%). The incorrect dosage of drugs was found in children whose drugs were bought on the street. The drugs from the family pharmacy box (57.14%) and pharmacy (79.37%) were for the most part correctly used. Nevertheless, among the misused drugs, those from the family pharmacy box were the most represented (78/116, 67.24%). The medication dosing errors, drug interactions and contraindications were also found in the “family pharmacy box” and «pharmacy».

## Discussion

After parents/guardians' interview, 295 hospitalized children were included and most of them were males (56%). Parents/guardians were mostly women (94%), unemployed or working in the informal sector and 85% of female guardians had at least one level of secondary education. This high proportion of women in our population of guardians/parents is explained by the fact that they are the ones who go to the health facilities with their children in our context. Most children (83%) came from the both health districts of Douala 3^rd^ and 5^th^while only 3% of the children resided in the health district of Douala 1^st^ where the LDH was located. This hospital is a referral hospital in the health district of Douala 1^st^, which hosts many referrals from other health facilities because of its superior technical platform and affordable care costs. The CPDH is a district hospital of 3^rd^ category located in the bordering part between Douala 5^th^ and Douala 3^rd^. The therapeutic itinerary was longer for patients included at the LHD compared to those from CPDH. The LHD is a central level structure with a higher technical platform and therefore able to accommodate patients with therapeutic failure or serious pathologies. Furthermore, most of parents (44.74%) had taken one to two days to see a health professional in our study. The mean time to go to a health facility was 2.9 days for boys compare to 2.4 days for girls and there was twice as many therapeutic remedies for boys. These results are similar to those found by Kabore et al. in Burkina Faso, where 69.7% of parents/guardians had taken the same time [35]. The long way in the therapeutic itinerary is responsible for the health management delay and this is detrimental to the child's survival. SM is considered as an important aspect of primary health care and desirable in most countries though can be dangerous [36,37]. Although the prevalence of self-medication in our population (more than 70% in 1^st^therapeutic recourse) was higher than that reported in other studies (from 38.5% in Germany to 56% in India), it remained the most widely used therapeutic itinerary by parents to treat their daughter's illness [25,38,39]. The four mean reasons reported by parent/guardians for the use of SM as first therapeutic itinerary in 1^st^therapeutic recourse was that they considered the disease as mild in 63.4%, they reported knowledge of treatment in 11.7% and the financial problem in 6.9%. A study conducted in Congo Lubumbashi has found that 31.5% of mothers practiced self-medication because of limited resources [40].

In India, minor illness (30.6%) and financial problems (12%) were the main reasons for the use of SM [41]. The most symptoms reported for the use of SM were fever, diarrhea, vomiting and cough as found by Atanda et al. in Congo, Kabore et al. in Burkina Faso and Togoobaataron et al. in Mongolia self-medicate [24,35,41]. Seventy-five of the two hundred and twenty-nine parents/guardians who did not go directly to a health facility used it as a second line remedy. The persistence or the severity of the disease was the main justifications given. For those who did not use health facility as a second resort, the Minor illness was the main reason evoked. The most commonly used drugs were antipyretics (46.6%), antimalarials (15%), antibiotics (10.2%) and antihelminthics (5.5%), these were related to common childhood infectious disease in our context. This is in line with results obtained in previous studies reported by Tsifiregna et al. in Madagascar, Kaushalet al. and Chakraborty et al. in India [25,36,42]. The drugs according to legal classification accounted for one-third of all drugs administrated to children and pharmaceutical medications without medical advice for this did not conform to the WHO recommendations on responsible self-medication [43]. A significant difference in misuse was observed between parents/guardians who reported reading the medications instructions and those who said they did not read it. These results are comparable to those reported by Kassabi-Borowiec et al, the reading of the leaflet was mentioned in 30% of respondents as the guiding element in taking drugs [27]. More than 60% of drugs consumed before admission to the hospital came from the family pharmacy box. Previous study has shown that non-prescription use was positively associated with keeping antibiotics at home and self-medication with antibiotics [42,44]. The drugs stored in the family medicine boxes can be explained by excessive prescription and/or non-compliance by the patient. It is therefore essential that doctors prescribe medications appropriately (quantity the patient needs) and encourage them to get rid of the remaining medications.

**Limitations:** First hospital study that gives us interesting results on the behavior of the parents, the therapeutic itineraries taken, and the specific drugs used and their origin in case of illness of their sick child. But this study was carried out in only 2 hospitals in Douala in urban areas and hence is not representative in pediatrics general population even through these are major hospitals at two different pyramidal levels in Cameroon. The results may underestimate the demand for traditional care. Also, it would have been relevant to extend the field of study to rural areas. In addition, the ab-sence of the variable socialization medium prevented the control results related to ethnicity.

## Conclusion

The response of many families to the use of medications without medical prescription/advice for their children's disease was self-medication before medical consultation. The inappropriate use of medications was more associated with the use of remaining drugs prescribed during prior illness and available from the family pharmacy box and lack of reading of the information leaflet drugs. Parent education could improve responsible self-medication, especially for children.

### What is known about this topic

- In most African countries, the combined use of modern medicine and alternative medicines prior to arrival to hospital is frequent;
- Several factors have been identified as determinants of these treatment options;
- Only two studies of care seeking behavior and household medications, were conducted in 2006 and 2011 based on data from the 2011 DHS-MICS.

### What this study adds

- Our study allowed us to have current data on the therapeutic approaches used by parents of sick children in a resource limited setting (self-medication was the most commonly used TR in 74% of patients);
- The use of health services is driven by perceived severity of the disease state;
- The drugs most commonly used for self-medication prior to hospitalization were antipyretic (46.6%) followed by antimalarial (15%) and antibiotics (10.2%). Of the drugs misused, dosage errors were observed in most cases, with fewer drug interactions and contraindications.

## Competing interests

The authors declare no competing interests.
